# The prognostic value of combined uric acid and neutrophil-to-lymphocyte ratio in acute ischemic stroke patients treated with intravenous thrombolysis

**DOI:** 10.1186/s12883-024-03628-w

**Published:** 2024-05-31

**Authors:** Chentao Wang, Meili Zhou, Tingting Kang, Shoujiang You, Yongjun Cao, Weina Kong, Jijun Shi

**Affiliations:** 1 Department of Neurology, The Second Affiliated Hospital of Soochow University, Suzhou, Jiangsu Province 215004 China; 2 Department of Neurology, The Nuclear Industry 417 Hospital, Xi’an, Shanxi Province 710600 China; 3 Clinical Research Center of Neurological Disease, The Second Affiliated Hospital of Soochow University, Suzhou, Jiangsu Province 215004 China

**Keywords:** Uric acid, NLR, Acute ischemic stroke, Thrombolysis, Prognosis, Recombinant tissue plasminogen activator (rt-PA)

## Abstract

**Background:**

Serum uric acid (UA) and the neutrophil-to-lymphocyte ratio (NLR) have been reported to be associated with outcomes in acute ischemic stroke (AIS). However, whether UA is related to the prognosis of AIS patients undergoing intravenous thrombolysis (IVT) remains inconclusive. We sought to explore the combined effect of UA and NLR on the prognosis of AIS treated with IVT.

**Methods:**

A total of 555 AIS patients receiving IVT treatment were enrolled. Patients were categorized into four groups according to the levels of UA and NLR: LNNU (low NLR and normal UA), LNHU (low NLR and high UA), HNNU (high NLR and normal UA), and HNHU (high NLR and high UA). Multivariable logistic regression analysis was used to evaluate the value of serum UA level and NLR in predicting prognosis. The primary outcomes were major disability (modified Rankin scale (mRS) score 3–5) and death within 3 months.

**Results:**

After multivariate adjustment, a high NLR (≥ 3.94) increased the risk of 3-month death or major disability (OR, 2.23; 95% CI, 1.42 to 3.55, *p* < 0.001). However, there was no statistically significant association between a high UA level (≥ 313.00 µmol/L) and clinical outcome. HNHU was associated with a 5.09-fold increase in the risk of death (OR, 5.09; 95% CI, 1.31–19.83; *P* value = 0.019) and a 1.98-fold increase in the risk of major disability (OR, 1.98; 95% CI 1.07–3.68; *P* value = 0.030) in comparison to LNNU.

**Conclusions:**

High serum UA levels combined with high NLR were independently associated with 3-month death and major disability in AIS patients after IVT.

## Introduction

Acute ischemic stroke (AIS) remains the second leading cause of both disability and death worldwide. Intravenous thrombolysis (IVT) with recombinant tissue plasminogen activator (rt-PA) is considered to be the most effective medical reperfusion treatment within 4.5 h of symptom onset in AIS patients. However, the prognosis of patients with AIS after IVT may be affected by modifiable factors such as C-reactive protein, white blood cell count and neutrophil-lymphocyte ratio (NLR) [1–4]. Hence, useful biomarker detection is essential for early risk assessment and effective treatment after IVT.

Uric acid (UA), the end product of purine metabolism in humans, is a major endogenous antioxidant with neuroprotective effect in the blood and an easily detectable and reliable biomarker in clinical practice [5]. However, it can also act as a pro-oxidant depending on the chemical microenvironment [6]. The role of UA in the prognosis of stroke is also conflicting [7]. With respect to preclinical studies, when serum UA levels were elevated, reduced brain damage and improved functional outcome were shown in a transgenic mouse (UOX+/-) model of focal ischemic stroke [8]. Moreover, a systematic review and meta-analysis of rodent data showed that UA significantly reduced infarct size and neurofunctional deficits [9]. With regard to AIS patients, the tertiary analysis of the URICO-ICTUS trial suggested that the combination of rt-PA and UA may prevent early ischemic deterioration [10]. In addition, a low serum UA level at Days 3 and 4 of onset was reported to be negatively associated with DWI volume at diagnosis [11]. Nevertheless, a retrospective study suggested that a high level of UA [≥ 340 µmol/L (5.712 mg/dl)] was related to stroke recurrence in older patients [12]. Furthermore, a U-shaped relationship between UA and functional outcomes in patients with AIS was found by Zhang and colleagues [13]. Patients with higher serum UA levels (> 380 µmol/L) or lower serum UA levels (≤ 250 µmol/L) were more likely to have a poor outcome compared to the baseline group (UA level 316–380 µmol/L). Some prospective cohort studies demonstrated that higher serum uric acid levels were associated with 3-month better functional outcome in AIS patients with IVT [14, 15]. Notably, a meta-analysis showed that there was no significant correlation between serum UA levels and the prognosis of AIS [16]. Thus, it is important to clarify the relationship between UA and the prognosis of AIS patients undergoing IVT [7, 17]. The inflammatory response plays an essential role in the pathophysiology of AIS. After AIS, the number of circulating neutrophils increases while the number of lymphocytes decreases, leading to an increased NLR. The NLR has become a relatively popular marker of inflammation. Previous studies have shown that the admission NLR could be a predictor of major disability and mortality [18]. A recent study demonstrated that the Day 1 NLR was better than the admission NLR as a biomarker for predicting AIS outcome after reperfusion treatment [19]. An additive interaction between serum UA and NLR in ischemic stroke recurrence was found in a population study [12]. Consistent with the results, the positive additive interaction between UA and NLR was also observed in cardiac death plus reinfarction for coronary artery disease patients [20]. However, the relationship between the combined effect of UA and NLR and the prognosis of AIS patients after IVT is not clear.

In the present study, we examined whether the serum UA level was associated with prognosis and further investigated the combined effect of UA and NLR on outcomes in AIS patients after IVT.

## Materials and methods

### Study participants

From May 2010 to May 2017, we consecutively enrolled AIS patients treated with intravenous rt-PA (Boehringer Ingelheim, Germany) within 4.5 h of onset and who were from the Soochow Stroke Registry system at our comprehensive stroke center in Suzhou, China. rt-PA was used according to the specific guidelines. The methods of participant enrollment have been described previously [2]. The exclusion criteria were as follows: an unclear time of symptom onset, diagnosis of a tumor, stroke mimics and incomplete clinical data. Finally, a total of 555 AIS patients receiving rt-PA treatment were retrospectively enrolled. Patients receiving intravenous rt-PA plus mechanical thrombectomy were not enrolled in the study. 28 patients who underwent surgery for acute brain edema were also not included in the study. Routine antithrombotic agents and additional medications were given as needed 24 h after thrombolysis, as described in our previous publication [2]. The study protocol was approved by the Ethics Committee of the Second Affiliated Hospital of Soochow University(JD-HG-2023-81), and informed consent was obtained from all participants or patient care providers.

### Data collection

Demographic characteristics, lifestyle risk factors, medical history, clinical laboratory tests and imaging (computed tomography and magnetic resonance imaging) were collected at the time of enrollment. A standard questionnaire was administered by trained staff to obtain all this information. The National Institutes of Health Stroke Scale (NIHSS) and the modified Rankin Scale (mRS) were used to assess stroke severity by trained neurologists. NIHSS scores at admission and 24 h after IVT were obtained, and mRS scores at admission and 3 months were obtained. According to the Trial of Org 10,172 in the Acute Stroke Treatment (TOAST) criteria, stroke etiology was determined based on a review of all medical records during hospitalization. All serum biochemical parameters were measured at admission using an Olympus Au5400 automatic biochemical analyzer (First Chemical Co., Ltd., Japan).

### Outcome assessment

The primary outcome was defined as the composite of death or major disability (mRS score 3–6) at the 3-month follow-up. The secondary outcomes included major disability (mRS score 3–5), odds of a 1-unit higher mRS and death. Deaths were reported by family members and/or were obtained from death certificates and medical records. The causes of death(neurological) in the study sample included cerebral hernia (28 patients), symptomatic intracranial hemorrhage (6 patients) and ischemic stroke (10 patients). The cause of death (non-neurological) in the study sample included myocardial infarction (3 patients), heart failure(3 patients), pneumonia(9 patients).

### Statistical analysis

To evaluate the combined effect of UA and NLR on the prognosis of AIS treated with IVT, we divided the study subjects into four groups: those with a UA level lower than the median and an NLR level lower than the median; those with a UA level lower than the median and an NLR level higher than the median; those with a UA level higher than the median and an NLR level lower than the median; and those with a UA level higher than the median and an NLR level higher than the median. Continuous variables were expressed as the mean ± standard deviation (SD) or as the median (interquartile range, IQR) and were compared using analysis of variance or the Wilcoxon rank-sum test. Categorical variables were expressed as the frequency (percent) and were compared using the chi-square test. Odds ratios (ORs) and 95% confidence intervals (95% CIs) were calculated by using a logistic regression model. Potential covariates such as age, sex, time from onset to thrombolysis, current smoking, alcohol consumption, admission NIHSS score, systolic blood pressure, plasma glucose, history of hyperlipidemia, hypertension, coronary heart disease, stroke, diabetes mellitus, and stroke subtypes were adjusted for in the multivariate model. A sensitivity analysis was performed to test the robustness of our findings. The continuous and categorical net reclassification index (NRI) and integrated discrimination improvement (IDI) were calculated to evaluate the predictive value of adding the combination of NLR and UA to conventional risk factors. A 2-sided *p* value < 0.05 was established as the level for statistical significance. All analyses were conducted using SAS statistical software (version 9.4, Cary, North Carolina, USA).

## Results

### Characteristics of the study population and clinical outcomes for binary of UA and NLR

The characteristics of the study patients are presented in Table 1. The patients with both high NLR and UA (HNHU group) accounted for 25% of the total studied participants. In comparison to the LNNU group, the HNHU group was more likely to be older and have higher admission NIHSS and mRS scores. In addition, HNHU patients differed in sex, current smoking, admission systolic blood pressure and metabolic profile. After adjusting for age, sex, time from onset to thrombolysis, current smoking, alcohol consumption and other covariates, a high NLR (≥ 3.94) was found to be an independent predictor of death or major disability (OR, 2.23; 95% CI, 1.42 to 3.55, *p* < 0.001; Table 2). Similar associations between a high NLR (≥ 3.94) and secondary outcomes are shown in Table 2. However, no statistically significant association between high UA level (≥ 313.00 µmol/L) and clinical outcome was detected.

**Table 1:** Characteristics of the participants according to NLR and UA among AIS patients after IVT

Characteristics^a^	NLR < 3.94/ UA < 313.00 µmol/L	NLR < 3.94/ UA ≥ 313.00 µmol/L	NLR ≥ 3.94/ UA < 313.00 µmol/L	NLR ≥ 3.94/ UA ≥ 313.00 µmol/L	*P* value
Patients, n	139	139	138	139	
Age, y	64.02 ± 11.94	63.61 ± 12.58	66.62 ± 12.45	67.35 ± 12.75	0.026
Male sex, n (%)	71(51.08)	105(75.54)	74(53.62)	103(74.10)	< 0.001
Current cigarette smoking, n (%)	42(30.22)	66(47.48)	40(28.99)	55(39.57)	0.004
Current alcohol drinking, n (%)	29(20.86)	31(22.30)	19(13.77)	23(16.65)	0.234
Time from onset to thrombolysis, h	3.00(2.62–3.70)	3.00(2.33–3.97)	3.33(2.67-4.00)	3.38(2.67-4.00)	0.081
Admission systolic BP, mm Hg	147.60 ± 19.10	147.27 ± 18.21	154.36 ± 23.36	148.44 ± 22.35	0.015
Admission diastolic BP, mm Hg	81.95 ± 12.83	82.24 ± 13.48	82.36 ± 15.15	83.78 ± 15.24	0.718
Triglycerides, mmol/L	1.07(0.85–1.44)	1.21(0.92–1.80)	1.09(0.79–1.38)	1.09(0.80–1.45)	0.014
Total cholesterol, mmol/L	4.54(3.89–5.27)	4.86(4.23–5.51)	4.52(3.81–5.22)	4.41(3.88–5.11)	0.008
LDL cholesterol, mmol/L	2.88(2.15–3.34)	3.04(2.47–3.65)	2.78(2.20–3.21)	2.70(2.22–3.27)	0.006
HDL cholesterol, mmol/L	1.16(0.95–1.38)	1.12(0.94–1.33)	1.21(1.02–1.50)	1.17(1.00-1.35)	0.049
Plasma glucose, mmol/L	6.70(5.60–8.60)	6.70(5.40–8.20)	7.35(6.40–9.30)	7.10(5.90–8.90)	< 0.001
Admission NIHSS score	8(4–12)	8(5–12)	12(7–18)	14(9–19)	< 0.001
Admission mRS score	4(3–5)	4(3–5)	5(4–5)	5(4–5)	< 0.001
History of hypertension, n (%)	99(71.22)	101(72.66)	111(80.43)	115(82.73)	0.058
History of hyperlipidemia, n (%)	58(41.73)	62(44.60)	40(28.99)	41(29.50)	0.008
History of diabetes mellitus, n (%)	26(18.71)	18(12.95)	31(22.46)	22(15.83)	0.190
History of stroke, n (%)	19(13.67)	22(15.83)	26(18.84)	25(17.99)	0.656
History of coronary heart disease, n (%)	6(4.32)	4(2.88)	5(3.62)	8(5.76)	0.665
History of atrial fibrillation, n (%)	34(24.46)	38(27.34)	51(36.96)	76(54.68)	< 0.001
Use of antihypertensive medications, n (%)	8(5.76)	10(7.19)	10(7.25)	16(11.51)	0.316
Stroke subtypes (TOAST)					< 0.001
Large artery atherosclerosis	69(49.64)	66(47.48)	52(37.68)	45(32.37)	
Small vessel occlusion	35(25.18)	37(26.62)	55(39.86)	76(54.68)	
Cardioembolism	28(20.14)	34(24.46)	21(15.22)	10(7.19)	
Stroke of other determined etiology	5(3.60)	0(0.00)	7(5.07)	6(4.32)	
Stroke of undetermined etiology	2(1.44)	2(1.44)	3(2.17)	2(1.44)	

^a^ continuous variables are expressed as the mean ± SD or median (IQR). Categorical variables are expressed as frequency (percent).

**Table 2:** ORs and 95% CI of clinical outcomes for binary of UA and NLR among AIS patients after IVT

		Multivariable adjusted		
	Cases(%)	OR	95% CI	*P* value
Primary outcome: death or major disability (mRS 3–6)				
UA ≥ 313.00 µmol/L^a^	115(41.37)	1.45	0.92–2.83	0.113
NLR ≥ 3.94^b^	149(53.79)	2.23	1.42–3.55	< 0.001
Secondary outcomes: major disability (mRS 3–5)				
UA ≥ 313.00 µmol/L^a^	85(30.58)	1.18	0.78–1.81	0.435
NLR ≥ 3.94^b^	105(37.91)	1.69	1.09–2.63	0.020
Modified Rankin scale^c^				
UA ≥ 313.00 µmol/L^a^	-	1.09	0.80–1.50	0.580
NLR ≥ 3.94^b^	-	2.17	1.55–3.03	< 0.001
Death				
UA ≥ 313.00 µmol/L^a^	30(10.79)	1.51	0.76–3.02	0.423
NLR ≥ 3.94^b^	44(15.88)	2.83	1.21–6.61	0.016

^a^ Adjusted for age, sex, time from onset to thrombolysis, current smoking, alcohol drinking, admission NIHSS score, systolic blood pressure, plasma glucose, and history of hyperlipidemia, hypertension, coronary heart disease, stroke, and diabetes mellitus, stroke subtypes and NLR (Binary).

^b^ Adjusted for age, sex, time from onset to thrombolysis, current smoking, alcohol drinking, admission NIHSS score, systolic blood pressure, plasma glucose, and history of hyperlipidemia, hypertension, coronary heart disease, stroke, and diabetes mellitus, stroke subtypes and UA (Binary).

^c^ odds of a 1-unit higher modified Rankin score.

### The association of NLR and UA with clinical outcomes

The association of NLR and UA with clinical outcomes among AIS patients after IVT is presented in Table 3. In the multivariate analysis, the HNHU group demonstrated a 5.09-fold increase in the risk of death (95% CI 1.31–19.83; *P* value = 0.019; Table 3) in comparison to the LNNU group after multivariate adjustment. Similarly, the HNHU group exhibited a 1.98-fold increase in the risk of major disability (95% CI 1.07–3.68; *P* value = 0.030; Table 3). However, the HNNU group was not linked to death (OR, 3.72; 95% CI 0.93–14.87; *P* value = 0.064; Table 3) or major disability (OR, 1.60; 95% CI 0.87–2.94; *P* value = 0.128; Table 3).

**Table 3:** Combined effects of NLR and UA on clinical outcomes among AIS patients after IVT

	Model 1				Model 2		
	OR	95%	*P* value		OR	95%	*P* value
Primary outcome: death or major disability (mRS 3–6)							
NLR < 3.94/UA < 313.00 µmol/L	1.00	-			1.00	-	
NLR < 3.94/UA ≥ 313.00 µmol/L	1.43	0.79–2.59	0.239		1.35	0.70–2.61	0.369
NLR ≥ 3.94/UA < 313.00 µmol/L	3.10	1.76–5.47	< 0.001		1.11	1.11–3.99	0.022
NLR ≥ 3.94/UA ≥ 313.00 µmol/L	5.74	3.19–10.33	< 0.001		1.67	1.67–6.23	< 0.001
Secondary outcomes: major disability (mRS 3–5)							
NLR < 3.94/UA < 313.00 µmol/L	1.00	-			1.00	-	
NLR < 3.94/UA ≥ 313.00 µmol/L	1.18	0.64–2.17	0.596		1.12	0.59–2.10	0.737
NLR ≥ 3.94/UA < 313.00 µmol/L	2.07	1.16–3.68	0.014		1.60	0.87–2.94	0.128
NLR ≥ 3.94/UA ≥ 313.00 µmol/L	3.01	1.68–5.40	< 0.001		1.98	1.07–3.68	0.030
Modified Rankin scale^a^							
NLR < 3.94/UA < 313.00 µmol/L	1.00	-			1.00	-	
NLR < 3.94/UA ≥ 313.00 µmol/L	1.12	0.72–1.73	0.615		0.99	0.63–1.54	0.950
NLR ≥ 3.94/UA < 313.00 µmol/L	2.77	1.79–4.30	< 0.001		1.96	1.25–3.08	0.003
NLR ≥ 3.94/UA ≥ 313.00 µmol/L	4.53	2.88–7.12	< 0.001		2.38	1.49–3.80	< 0.001
Death							
NLR < 3.94/UA < 313.00 µmol/L	1.00	-			1.00	-	
NLR < 3.94/UA ≥ 313.00 µmol/L	2.21	0.52–9.36	0.284		2.14	0.47–9.81	0.328
NLR ≥ 3.94/UA < 313.00 µmol/L	5.29	1.45–19.31	0.012		3.72	0.93–14.87	0.064
NLR ≥ 3.94/UA ≥ 313.00 µmol/L	8.77	2.43–31.61	< 0.001		5.09	1.31–19.83	0.019

Model 1, adjusted for age, sex, time from onset to thrombolysis, current smoking, alcohol drinking, systolic blood pressure, plasma glucose, and history of hyperlipidemia, hypertension, coronary heart disease, stroke, and diabetes mellitus, and stroke subtypes.

Model 2, further adjusted for admission NIHSS score.

^a^ odds of a 1-unit higher modified Rankin score.

### Reclassification and discrimination statistics for clinical outcomes by combination of NLR and UA

Table 4 shows whether adding the combination of NLR and UA to conventional risk factors improved the risk prediction of prognosis. Adding the combination of NLR and UA to the conventional model significantly improved the discriminatory power for death or major disability (continuous net reclassification index 43.76%; integrated discrimination improvement 2.04%), major disability (continuous net reclassification index 27.17%) and death (continuous net reclassification index 58.16%; integrated discrimination improvement 1.89%).

**Table 4:** Reclassification and discrimination statistics for clinical outcomes by combination of NLR and UA among AIS patients after IVT

	Continuous NRI, %			Categorical NRI^a^, %			IDI, %	
	Estimate (95% CI)	*p* value		Estimate (95% CI)	*p* value		Estimate (95% CI)	*p* value
Death or major disability(mRS score 3–6)								
Conventional model	Reference			Reference			Reference	
Conventional model + combination of NLR and UA^b^	43.76(27.11–60.40)	< 0.001		4.68(-0.58-9.94)	0.081		2.04(0.84–3.24)	< 0.001
Major disability(mRS score 3–5)								
Conventional model	Reference			Reference			Reference	
Conventional model + combination of NLR and UA^b^	27.17(9.06–45.27)	0.003		1.12(-3.34-5.58)	0.624		0.95(0.01–1.85)	0.039
Death								
Conventional model	Reference			Reference			Reference	
Conventional model + combination of NLR and UA^b^	58.16(31.62–84.70)	< 0.001		6.96(-5.27-19.2)	0.265		1.89(0.29–3.48)	0.020

The conventional model included age, sex, time from onset to thrombolysis, current smoking, alcohol drinking, systolic blood pressure, plasma glucose, and history of hyperlipidemia, hypertension, coronary heart disease, stroke, and diabetes mellitus, and stroke subtypes.

^a^ Patients were divided into 3 risk categories: <5%, 5-15%, and > 15%.

^b^ combination of NLR and UA was divided into four groups: NLR < 3.94/UA < 313.00 µmol/L, NLR < 3.94/UA ≥ 313.00 µmol/L, NLR ≥ 3.94/UA < 313.00 µmol/L, and NLR ≥ 3.94/UA ≥ 313.00 µmol/L

## Discussion

Our study showed that concurrent high NLR and high serum UA levels are associated with increased risks of 3-month major disability and death. But there is no significant correlation between serum UA level and the prognosis of AIS patients with IVT.

An observational study showed that each milligram per deciliter increase in serum UA was associated with a 12% increase in the odds of good clinical outcome in patients with AIS [21]. Subsequently, in a rat model of thromboembolic stroke, exogenous administration of UA extended the benefits of rt-PA [22]. Likewise, a pilot trial showed that an infusion of 1 g UA following rt-PA reduced circulating biomarkers related to poor clinical outcomes without serious adverse effects [23]. In a subsequent clinical study, an intravenous infusion of 1 g UA was given in combination with rt-PA infusion. The proportion of patients with excellent outcome (an mRS score of 0–1, or an mRS score of 2 if the premorbid mRS was 2, at 90 days) was not significantly different between the UA group and placebo group [24]. The reanalysis of the clinical trial revealed that UA administration significantly elevated the proportion of excellent outcomes in women but not in men and that UA may prevent early ischemic stroke progression [10]. However, serum UA levels were not measured in that clinical trial, resulting in an unknown magnitude and duration of UA elevation following UA administration. A recent study showed that a high baseline UA level was positively associated with a good 3-month outcome in acute ischemic stroke patients with reperfusion therapy [25]. According to baseline UA levels, patients were classified into three tertiles: T1 (123–303 µmol/L), T2 (304–385 µmol/L) and T3 (385–704 µmol/L). The grouping method and selection of the study population may explain the inconsistent findings between our study and the study above. Therefore, more studies are needed to further investigate the relationship between serum UA and the prognosis of AIS patients after IVT.

The NLR is significantly associated with clinical outcomes in AIS. Meta-analyses have shown that an elevated NLR is correlated with an increased risk of ischemic stroke, unfavorable functional outcome at 3 months and increased mortality [26]. Our results showed that a high NLR level was an independent predictor for poor clinical outcome in AIS after IVT, and this is in line with our previous study [2].

The correlation between UA and NLR has gained substantial attention recently. One study showed that the NLR was positively correlated with UA in patients with chronic kidney disease [27]. However, in a study on multiple sclerosis, a negative correlation was observed between the NLR and UA [28]. The findings of the same study indicated that combined evaluation of NLR and UA may be a more effective approach in determining disability in patients with multiple sclerosis than assessing these parameters separately. These studies showed that there is a correlation between UA and the NLR, but this correlation may vary in different diseases. Previous studies have demonstrated that both UA and NLR are closely related to AIS, but few studies have examined UA, NLR, and AIS together. A recent study suggested that patients with high UA and high NLR levels are at greater risk for AIS recurrence [12]. Our study explored the combined effect of NLR and UA on the outcomes of AIS patients after IVT, and we found that high NLR and serum UA levels are associated with increased risks of 3-month major disability and death. The possible mechanisms are oxidation and inflammation.

In general, the reference interval of serum UA is 1.5 to 6.0 mg/dl in women and 2.5 to 7.0 mg/dl in men. Hyperuricemia is defined as a serum UA level greater than 6.0 mg/dL in women and 7.0 mg/dL in men [29]. Only suitable concentrations of serum UA may have a protective effect. In our study, the demarcation point of UA was 313 µmol/L (5.3 mg/dl). One dose‒response study showed a J-shaped trend between ascending UA levels and a higher risk of suffering from ischemic stroke. When the UA reached 5.25 mg/dl, it started to become statistically significant [30]. Therefore, it is reasonable to speculate that the high level of UA (> 5.3 mg/dl) acts as a pro-oxidant. A previous experimental study showed that increased UA (> 6 mg/dl) was associated with endothelial dysfunction and increased oxidative stress [31].

UA can also induce inflammation. A population study reported a positive relationship between serum UA and acute-phase reactants, such as C-reactive protein, fibrinogen and complement C3 [32]. The same study also examined the effect of UA on the expression of inflammatory biomarkers in vitro and found that hyperuricemia might induce inflammation by activating the proinflammatory NF-κB signaling cascade. Another study found that hyperuricemia (up to 50 mg/dL) can exacerbate chronic inflammation by altering the balance of interleukin-1β/interleukin-1Ra [33]. Moreover, elevated UA (9 mg/dl) was reported to induce vascular inflammation by upregulating the (pro) renin receptor in human umbilical vein endothelial cells [34]. Inflammatory biomarkers such as C-reactive protein and fibrinogen are upregulated following AIS [2]. The correlations of these biomarkers with increased mortality and poor functional outcome of stroke have been reported. Thus, we speculate that high UA may exacerbate poor prognosis in AIS patients with a high NLR through oxidation and inflammation.

UA and NLR were easily accessible biomarkers of oxidative stress and inflammation from daily blood examinations and can be easily translated into clinical practice. To date, no studies have investigated the relationship between the combined effect of UA and NLR and the outcomes of AIS patients after IVT. This is the innovative point and a major strength of this research. However, several limitations should also be noted. First, this study was a single-center study, and the population was relatively small, which may limit the generalization of our findings. Second, a subgroup analysis with stratification by sex was not performed to assess the effect of UA on the prognosis of AIS patients after IVT. Third, UA and NLR levels were only measured at admission. Without consecutive measurements, we have no data to examine the correlation between UA and NLR variations and stroke prognosis. Last, A clinical study showed that cerebral infarcts in the territory of the anterior cerebral artery have a better prognosis than infarcts in the territory of the middle cerebral artery [35]. It is reasonable to investigate the effect of the combination of uric acid and neutrophil-to-lymphocyte ratio on the different vascular cerebral topographies. Since cerebral infarcts in the ACA territory were infrequent, there was not a sufficient sample size in our study to conduct tratified analysis of different infarcted areas. Future studies are necessary to explore the effect of the combination of uric acid and neutrophil-to-lymphocyte ratio on the different vascular cerebral topographies.

## Conclusion

There might be no significant association between UA and the prognosis of AIS patients with IVT. The combination of high UA and high NLR may be a predictor of 3-month death and major disability in AIS patients with IVT. This study suggests that strict control of UA and inflammatory parameters may help to improve outcomes for patients with ischemic stroke.

## Data Availability

The datasets used and analysed during the current study are available from the corresponding author on reasonable request.

## Abbreviations

UA: Serum uric acid
NLR: Neutrophil-to-lymphocyte ratio
AIS: Acute ischemic stroke
rt-PA: Recombinant tissue plasminogen activator
IVT: Intravenous thrombolysis
NIHSS: National Institutes of Health Stroke Scale
Mrs: Modified Rankin Scale
TOAST: Trial of Org 10172 in the Acute Stroke Treatment
SD: Standard deviation
IQR: Interquartile range
OR: Odds ratio
CI: Confidence interval
NRI: Net reclassification index
IDI: Integrated discrimination improvement
